# Complex Network Model Reveals the Impact of Inspiratory Muscle Pre-Activation on Interactions among Physiological Responses and Muscle Oxygenation during Running and Passive Recovery

**DOI:** 10.3390/biology11070963

**Published:** 2022-06-25

**Authors:** Fúlvia Barros Manchado-Gobatto, Ricardo Silva Torres, Anita Brum Marostegan, Felipe Marroni Rasteiro, Charlini Simoni Hartz, Marlene Aparecida Moreno, Allan Silva Pinto, Claudio Alexandre Gobatto

**Affiliations:** 1 Laboratory of Applied Sport Physiology, School of Applied Sciences, University of Campinas, Limeira 13484-350, Brazil; anita_brum@hotmail.com (A.B.M.); felipemarroni@hotmail.com (F.M.R.); cgobatto@unicamp.br (C.A.G.); 2 Department of ICT and Natural Sciences, Norwegian University of Science and Technology, 6009 Ålesund, Norway; ricardo.torres@ntnu.no; 3 Postgraduate Program in Human Movement Sciences, Methodist University of Piracicaba, Piracicaba 13400-000, Brazil; charlinih@hotmail.com (C.S.H.); marlene.moreno@unimep.br (M.A.M.); 4 Department of Sport Sciences, Faculty of Physical Education, University of Campinas, Campinas 13083-851, Brazil; allan.pinto@ic.unicamp.br; 5 Brazilian Synchrotron Light Laboratory, Brazilian Center for Research in Energy and Materials, Campinas 13083-970, Brazil

**Keywords:** acute inspiratory loading, performance, near-infrared spectroscopy (NIRS), muscle oxygenation, tethered running, computational modelling

## Abstract

**Simple Summary:**

Different warm-ups can be used to improve physical and sports performance. Among these strategies, we can include the pre-activation of the inspiratory muscles. Our study aimed to investigate this pre-activation model in high-intensity running performance and recovery using an integrative computational analysis called a complex network. The participants in this study underwent four sessions. The first and second sessions were performed to explain the procedures, characterize them and determine the individualized pre-activation intensity (40% of the maximum inspiratory pressure). Subsequently, on different days, the subjects were submitted to high-intensity tethered runs on a non-motorized treadmill with monitoring of the physiological responses during and after this effort. To understand the impacts of the pre-activation of inspiratory muscles on the organism, we studied the centrality metrics obtained by complex networks, which help in the interpretation of data in a more integrated way. Our results revealed that the graphs generated by this analysis were altered when inspiratory muscle pre-activation was applied, emphasizing muscle oxygenation responses in the leg and arm. Blood lactate also played an important role, especially after our inspiratory muscle strategy. Our findings confirm that the pre-activation of inspiratory muscles promotes modulations in the organism, better integrating physiological responses, which could increase performance and improve recovery.

**Abstract:**

Although several studies have focused on the adaptations provided by inspiratory muscle (IM) training on physical demands, the warm-up or pre-activation (PA) of these muscles alone appears to generate positive effects on physiological responses and performance. This study aimed to understand the effects of inspiratory muscle pre-activation (IM_PA_) on high-intensity running and passive recovery, as applied to active subjects. In an original and innovative investigation of the impacts of IM_PA_ on high-intensity running, we proposed the identification of the interactions among physical characteristics, physiological responses and muscle oxygenation in more and less active muscle to a running exercise using a complex network model. For this, fifteen male subjects were submitted to all-out 30 s tethered running efforts preceded or not preceded by IM_PA_, composed of 2 × 15 repetitions (1 min interval between them) at 40% of the maximum individual inspiratory pressure using a respiratory exercise device. During running and recovery, we monitored the physiological responses (heart rate, blood lactate, oxygen saturation) and muscle oxygenation (in vastus lateralis and biceps brachii) by wearable near-infrared spectroscopy (NIRS). Thus, we investigated four scenarios: two in the tethered running exercise (with or without IM_PA_) and two built into the recovery process (after the all-out 30 s), under the same conditions. Undirected weighted graphs were constructed, and four centrality metrics were analyzed (Degree, Betweenness, Eigenvector, and Pagerank). The IM_PA_ (40% of the maximum inspiratory pressure) was effective in increasing the peak and mean relative running power, and the analysis of the complex networks advanced the interpretation of the effects of physiological adjustments related to the IM_PA_ on exercise and recovery. Centrality metrics highlighted the nodes related to muscle oxygenation responses (in more and less active muscles) as significant to all scenarios, and systemic physiological responses mediated this impact, especially after IM_PA_ application. Our results suggest that this respiratory strategy enhances exercise, recovery and the multidimensional approach to understanding the effects of physiological adjustments on these conditions.

## 1. Introduction

Inspiratory muscles (IM) are essential for performing physical effort at different intensities [1], as well as for maintaining the quality of post-exercise recovery [2,3]. Aiming to improve respiratory muscle efficiency, specific respiratory muscle training has been the target of many investigations in sports performance [4,5,6,7,8] and has been used as a procedure to boost the physical capacity and physiological state of active individuals [9,10] and patients [11,12,13]. With long-term training, IM showed positive adaptations, such as the reduction of the respiratory metaboreflex mechanism, which contributes to the redirection of blood flow to more active muscles during physical effort [14,15].

Although several studies have focused on the adaptations provided by IM training, such as a significant resource to improve performance [6,14], the warm-up or pre-activation (PA) of these muscles already seems to generate positive effects on physiological responses [16,17] and performance [18,19]. So, this individualized and licit strategy has been highlighted as ergogenic for elite swimmers [19], badminton players [20], judo athletes [21] and runners [18]. On the other hand, the literature is scarce about the effects of inspiratory muscle pre-activation (IM_PA_) on non-athlete participants, revealing some positive effects on pulmonary function [16] and breathlessness sensations and an improvement in exercise tolerance [22] with inspiratory exercise at 40% of the maximal inspiratory pressure (MIP) before the main exercise.

Based on pre-activation concepts, it is probable that the IM warm-up using individual load flow restriction by a mechanical device (i.e., external mechanical loading) [23] acts as an important pre-activator of the organism, preparing it for high-intensity demands and recovery processes and boosting performance. In addition to studies that investigate electroencephalographic (EEG) activity prior to motor activation [24] and diaphragm motor-evoked potentials [25], the monitoring of physiological responses (e.g., HR, VO_2_ and SpO_2_) during effort and recovery seems to contribute to the analysis of the impact of IM_PA_ on the organism. Metabolically, lactate can develop a significant role during different processes, including exercise and recovery. Currently, it is considered a major energy source for mitochondrial respiration, the major gluconeogenic precursor and a signaling molecule [26]. This metabolite, produced during high-intensity exercise [27,28], can be used as an important fuel by different tissues, including less-active muscles after effort. Regarding IM warm-up, Lin et al. [20] observed a reduction in blood lactate accumulation during an incremental field test using this strategy. In this way, we strongly believe that the effects of IM_PA_ on high-intensity running effort and recovery can be related to blood lactate shuttle.

Furthermore, the analysis of muscle oxygenation by wearable near-infrared spectroscopy (NIRS) [29,30] offers the possibility of exploring O_2_ balance in skeletal muscle continuously, both during and after exercise. The NIRS technique has been applied successfully to measure the muscle oxygenation changes in a single muscle [31,32] and in two or more muscles [33,34,35,36,37]. Regarding the use of the NIRS analysis associated with the acute loading of IM on exercise, there are few investigations in the literature. In 2015, Ohya et al. [38] investigated the effects of IM warm-up on locomotor muscle oxygenation (vastus lateralis—VL) in healthy males submitted to cycling exercise. They did not observe significant performance enhancement or muscle oxygenation during high-intensity intermittent sprint cycling in untrained participants. Recently, Richard & Billaut [39] conducted an interesting study applying the IM warm-up to elite speed skaters before 3000 m time trials. Corroborating the results of Ohya et al. [38], no differences in speed performance nor in the tissue saturation index (TSI) and total hemoglobin (tHb) after an IM warm-up strategy were observed, at least in the VL muscle. However, investigations using portable NIRS in less and more active muscle concomitantly are scarce, but they can contribute strongly to the comprehension of the physiological effects promoted by IM_PA_.

The IM_PA_ seems to provoke positive effects on performance and physiological adjustments, but this is not the consensus in the literature. However, to the best of our knowledge, only one recent study conducted by our group involving this strategy is based on complex analysis—rather than only conventional statistics—to interpret these data [21]. In this case, by the complex network model, the positive impact of the IM_PA_ at 40% of the MIP was confirmed. So, it is possible that important physiological changes occur in athletes or non-athletes submitted to IM_PA_ prior to different exercise types but are not always detectable by statistical analyses based on “cause-effect” approaches. In previous studies, we have worked with more integrated models to investigate the physical exercise [40,41] and sports-related context [21,42,43], applying the concepts of complex networks [44,45] for biological data interpretation [46,47]. This line of investigation has contributed strongly to understanding biological responses that do not depend only on an isolated factor [46,47,48,49,50,51], which occurs with physiological adjustments in effort and recovery. In this way, we believe that this computational model can improve the knowledge about IM_PA_, especially by integrating analyses conducted with many responses during exercise and recovery. Basically, in complex network models, the centrality metrics that are returned from the graphs built for different scenarios are able to highlight, within a dataset, those which are the main players in an integrative context. Centrality measures are capable of quantifying the capacity of a node to influence or be influenced by other parameters in a connection topology [52]. Among the many available centrality metrics, we highlight here the analysis of the Degree, Betweenness, Eigenvector and Pagerank, each with a purpose in studies with complex networks [21,42,43,52,53,54,55,56].

Here, we investigated the impact of one mechanical IM_PA_ protocol (load at 40% of the MIP) on tethered running power on a non-motorized treadmill. Thus, by applying the concept of complex networks for integrative analysis among physiological responses, we aimed to understand the effects of this specific IM_PA_ on important physiological measures for effort and recovery, such as blood lactate, HR and muscle oxygenation in the biceps brachii (BB) and the vastus lateralis (VL), which are, respectively, less and more active in high-intensity running. We investigated four scenarios: two in the tethered running exercise (with or without IM_PA_) and two others built to understand the recovery process after the all-out 30 s (AO30) running effort under the same conditions. We hypothesize that the IM_PA_ presents changes in physiological responses in both exercise and recovery, and the complex network model is sensitive enough to detect the effects of this pre-activation strategy on physiological connections.

## 2. Methods

### 2.1. Subjects

Fifteen physically active young men participated in the study (23 ± 1 years, 73.2 ± 2.0 kg, 1.77 ± 0.02 m, 6.5 ± 0.5% body fat, 144.3 ± 10.2 cm H_2_O of MIP, mean and peak global strength index of IMs (S-Index) equal to 123.7 ± 3.7 and 139.1 ± 3.4 cm H_2_O, respectively). Firstly, the subjects answered the International Physical Activity Questionnaire (IPAQ), in which the minimum score required to classify them as “physically active” was used as the inclusion criterion [57]. All of the subjects reported no cardiovascular, respiratory, metabolic or orthopaedic disease and no use of drugs, ergogenic supplements or medications. This study was conducted in agreement within the ethical recommendations of the Declaration of Helsinki, and all experiments were approved by the Research Ethics Committee of The School of Medical Sciences (protocol number 99783318.4.0000.5404). The participants were only evaluated after having received information about the experimental procedures and risks and signing an informed consent form.

### 2.2. Experimental Design

**Figure 1:**
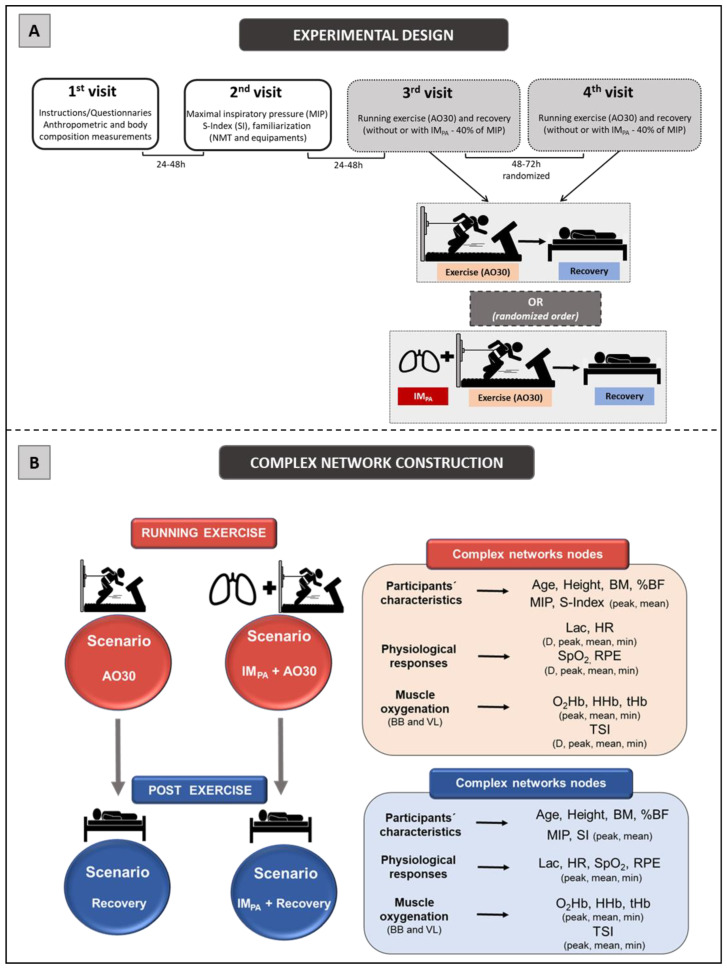
**Panel A.***Experimental design*. First and second visits were conducted for sample characterization and familiarization. Additionally, the maximal inspiratory pressure (MIP) and global strength index of inspiratory muscles (SI) were measured. Exercise (all-out in tethered running, AO30) and recovery (both, without or with inspiratory muscle pre-activation (IM_PA_)) were conducted at the third and fourth randomized visits, separated by 48–72 h. **Panel B.** Scenarios for constructing the complex networks and complex network nodes used in these four scenarios. **Legend:** AO30 = all-out 30 s, IM_PA_ = inspiratory muscle pre-activation, BM = body mass, %BF = percentage of body fat, MIP = maximal inspiratory pressure, SI = global strength index of inspiratory muscles, Lac = blood lactate, HR = heart rate, SpO_2_ = arterial oxygen saturation, RPE = perceived exertion, D = delta between final and initial results, BB = biceps brachii, VL = vastus lateralis, O_2_Hb = oxyhemoglobin relative concentration, HHb = deoxyhemoglobin relative concentration, tHb = total hemoglobin, TSI = tissue saturation index.

The experimental design consisted of four laboratory visits, separated by 24−72 h (Figure 1, Panel A). Firstly, the subjects received information about the experimental design and signed a consent form. They answered the IPAQ and a questionnaire for health characterization. Next, the participants were submitted to anthropometric and body composition measurements, including body mass (balance portable digital scale, with a maximum capacity of 150 kg and an accuracy of 100 g, Toledo^®^ model 2098, São Bernardo do Campo, Brazil) and height (by a stadiometer (Cescorf^®^, Porto Alegre, Brazil). To estimate the percentage of body fat (%BF), seven skinfold thicknesses of the right side of the body were evaluated (triceps, chest, subscapular, midaxillary, suprailiac, abdominal and thigh by the Lange^®^ skinfold caliper, Beta Technology, Santa Cruz, USA), and the equation proposed by Jackson & Pollock, following by Siri et al., was applied [58,59]. On the second day, the maximal inspiratory pressure (MIP) and S-Index (SI) were obtained (with a 1 h interval between them), and the tethered running familiarization was conducted on a non-motorized treadmill (NMT). The third and fourth days were randomly dedicated to AO30 in NMT, preceded or not preceded by the IM_PA_, aiming to determine the power in tethered running and physiological measurements in two conditions. In these sections, the subjects were equipped with two NIRS devices (one in BB and the other in VL) to acquire muscle oxygenation signals and with the HR monitor. After the running test, the participants remained at rest (18 min in the dorsal decubitus position) for the continuous monitoring of HR, SpO_2_ and muscle oxygenation responses. Additionally, blood samples and Borg scale scores [60] were collected at rest immediately after the effort and after each 2 min of passive recovery in order to assess blood lactate and perceived exertion (RPE). All of the trials were completed under controlled laboratory conditions at a similar temperature, relative humidity and luminosity.

### 2.3. Maximal Inspiratory Pressure and S-Index Determination

The maximal inspiratory pressure (MIP) measurement was obtained from residual volume in accordance with Hartz et al. [6]. The evaluation was conducted by a trained researcher who demonstrated the correct performance of the respiratory maneuver. The volunteers remained seated in a chair, wearing a nose clip and a plastic mouthpiece that was connected to an analogical manovacuometer (±300 cmH_2_O; GER-AR, São Paulo, SP, Brazil) used to measure maximal pressures. A small hole (2 mm) was introduced in the rigid mouthpiece in order to prevent glottic closure. The volunteers were instructed to complete three to five acceptable and reproducible maximal maneuvers (i.e., differences of 10% or less between values). Each inspiratory effort was sustained for at least 1 s, and the highest value reached was registered for further analysis. An interval of about 1 min was allowed between efforts [61].

The global strength index of IMs (S-Index, SI) was measured by the POWERbreathe^®^ device, model K5 (IMT Technologies Ltd., Birmingham, UK). The participants performed the assessment protocol in the orthostatic position with a nose clip. Thirty dynamic inspirations were performed slowly with verbal encouragement to inspire a greater air capacity [62]. During the protocol, the curves related to the inspirations were monitored by graphic records issued by the Breathe-link^®^ software (IMT Technologies Ltd., Birmingham, UK). At the end of the test, the algorithm provided the mean and maximum values of the SI (in units of cmH_2_O).

### 2.4. Inspiratory Muscle Pre-Activation

The IM_PA_ was characterized by 2 sets of 15 breaths with a 40% maximal inspiratory mouth pressure load (40% of the MIP) using the POWERbreathe inspiratory muscle trainer, model K5 (IMT Technologies Ltd., Birmingham, UK), with a 60 s rest between sets. The choice of flow restricted to 40% of the MIP was based on different studies that use this restriction, aiming to improve performance by mechanical breath warm-up [16,21,39].

### 2.5. High-Intensity Running Effort and Post-Exercise

As a high-intensity exercise, the participants performed a tethered run in the NMT ergometer specifications are detailed by Manchado-Gobatto et al. [34]). All of the procedures were similar in the third and fourth sessions, except for the application of the IM_PA_ in one of these. After arriving in the laboratory, the participants were equipped with two wearable NIRS devices and a heart rate monitor. They remained in a sitting position at rest for 3 min, and their baseline responses were registered. The first blood sample was collected from the ear lobe. After this, the participants warmed up for 5 min by running on a motorized treadmill (Inbramed Super ATL, Inbrasport, Porto Alegre, Brazil) with the intensity fixed at 7.0 km/h^−1^. After the warm-up, a 5 min passive recovery was taken to return the physiological responses to rest levels. The IM_PA_ was applied (or not) according to the session characteristics. After this, the running effort was applied for 30 s (AO30), and the mechanical, heart rate and muscle oxygenation responses were recorded. The participants received constant verbal encouragement during the tethered test exercise.

Immediately after the exercise, a pulse oximeter was placed on the subject’s finger, and blood capillary samples were extracted from the ear lobe into heparinized capillary tubes. With the aim of conducting recovery investigations, HR, arterial oxygen saturation (SpO_2_) and muscle oxygenation were monitored continuously for up to 18 min after AO30, with blood capillary samples extracted immediately and every 2 min during this recovery period.

### 2.6. Power in High-Intensity Tethered Exercise

The exercise protocols were conducted on a standardized non-motorized treadmill. As described by Gobatto et al. [42] and Manchado-Gobatto et al. [34], this ergometer is composed of an acquisition system consisting of a strain gauge (CSA/ZL-500 MK Control, Sao Paulo, Brazil), a portable amplifier (MKTC5-10, MK Control, Sao Paulo, Brazil) and an acquisition module (NI USB-6009, National Instruments, Austin, USA). The participants were submitted to AO30 tethered running using an inextensible steel cable tied to a system. In this structure, there was a load cell (CSL/ZL-500, MK Controle e Instrumentação Ltd., São Paulo, Brazil) to measure the horizontal force during running. Four load cells positioned under the NMT platform were capable of capturing the vertical force. An effect hall sensor, inserted in the frontal axis of the NMT, provided pulses for velocity acquisition. The product of the force and running velocity was considered to be the tethered running power. The signals of mechanical variables were captured at a 1000 Hz acquisition (LabView Signal Express 2009 National Instruments^®^, Austin, TX, USA). To maintain high system quality, the NMT was calibrated daily, modulated and subsequently transferred to MatLab (R2008a MatLab^®^, MathWorks, Natick, MA, USA). The peak (*p*), mean (m), and minimum (min) values of power were displayed in absolute (W) and relative body mass values (W·kg^−1^) with a MatLab routine. The fatigue index (FI = (peak power—minimum power)/peak power × 100) was also calculated. These values were used to compare the effects of the IM_PA_ on tethered running performance.

### 2.7. Muscle Oxygenation Measurements and Analyses

During all periods of the third and fourth visits, the changes in muscle oxygenation were assessed continually using the NIRS technique at wavelengths between 760 and 850 nm. For this, the NIRS wearable system (2 × *PortaMon*, Artinis Medical Systems, Elst, Netherlands) registered changes in the relative concentrations of oxyhemoglobin (O_2_Hb), deoxyhemoglobin (HHb) and total hemoglobin (tHb) and the values of the tissue saturation index—TSI (%) at a signal capture frequency of 10 Hz. Two NIRS devices were positioned on the medial biceps brachii (BB) portion [35,63] and vastus lateralis (VL) belly (15 cm above the proximal edge of the patella and 5 cm towards the external side [64], parallel to the long axis of the muscle), which were considered as less and more active during the running effort [34], respectively. The signal data were captured and smoothed using a 10th-order low-pass-zero phase Butterworth filter (cut-off frequency 0.1 Hz) using the *Oxysoft* (Artinis Medical System, Elst, Netherlands). To determine the peak, mean and minimum values of O_2_Hb, HHb and tHb, we considered the difference from the baseline data (final 30 s of the 3 min baseline period).

### 2.8. Arterial Oxygen Saturation, Heart Rate and Blood Lactate Concentration

Arterial oxygen saturation was captured at rest immediately after the AO30 exercise and during all recovery phases with a pulse oximeter (Oxifast Takaoka, São Paulo, Brazil). The heart rate was registered during all protocols (at 1 s intervals) by a heart rate monitor (Polar V800, Polar Electro Oy, Kempele, Finland). Blood samples (25 µL) were collected at rest and during the post-exercise phase from the ear lobe with a heparinized capillary. The blood samples were deposited into microtubes (Eppendorf, 1.5 mL) containing 50 µL of 1% sodium fluoride (NaF) and were subsequently frozen at −20 °C. The blood lactate concentrations were determined by a lactate analyzer (YSI-2300-STAT-Plus™, Yellow Springs, OH, USA).

### 2.9. Complex Network Construction: Scenarios and Centrality Metrics

The four scenarios constructed in this study were modelled considering the use of complex networks, two of them involving exercise results (AO30 and IM_PA_ + AO30) and the other two based on recovery responses (Recovery AO30 and Recovery IM_PA_ + AO30) (Figure 1, Panel B). In all cases, the analysis of these complexes was performed using the software Gephi (version 0.9.2, Gephi, Paris, France)—implemented in the JAVA programming language and applying the Fruchterman–Reingold layout [65]—after the data processing step by a specific algorithm constructed for this study in MatLab. The networks were built considering undirected weighted graphs G = (V, E, w) for our scenarios, where V represents the nodes, E represents the interaction between two physiological variables (edges) and w is the weight function [53]. In this case, the nodes of the graph correspond to the participant’s characteristics, physiological responses and muscle oxygenation parameters in BB and VL (see Figure 1, Panel B for more detail) in exercise and recovery scenarios. The undirected edges were pointed out by correlations among the nodes (participants´ characteristics, physiological responses and muscle oxygen) which were weighted by significant “r” values (*p* ≤ 0.05) according to Pearson’s correlation (linear regression test) in each scenario [42] (MatLab^®^,MathWorks, Natick, MA, USA).

The same 46 nodes were used to construct the graphs in the exercise scenarios (AO30 and IM_PA_ + AO30). Aiming to establish the recovery scenarios (recovery and IM_PA_ recovery), 52 nodes were included in these respective graphs. In this study, we used four centrality metrics to provide the complex network interpretation: Degree, Betweenness Centrality, Eigenvector Centrality and Pagerank. The use of these metrics was based on previous studies by our group in exercise investigations [21,40,41,42,43] and on Oldham et al. [52]. The methodological details about the metrics adopted here are visualized in Supplementary File S1-Complex network centrality metrics: Degree, Betweenness, Eigenvector, and Pagerank.

### 2.10. Statistical Analysis

The normality of the distributions and the homogeneity of the variance were initially tested by the Shapiro−Wilk and Levene tests, respectively. A paired Student’s *t*-test was applied to identify the differences between the performance in exercise without or with IM_PA_ (peak, mean and minimum running power), physiological responses (blood lactate, heart rate, arterial oxygen saturation) and muscle oxygenation (tissue saturation index in BB and VL) at rest and after efforts in two conditions. Statistical analyses were performed using the software STATISTICA^®^ (7.0 version) and MatLab^®^ (MathWorks, Natick, MA, USA). In all cases, the statistical significance was set at *p* ≤ 0.05.

## 3. Results

Table 1 shows the peak, mean and minimum values of the absolute running power (W) and running power (W·kg^−1^) relative to the body mass observed in the participants submitted to tethered running without (AO30) and with IM_PA_ (IM_PA_ + AO30) using a mechanical device with a 40% MIP load. Here, we also presented the main physiological results at rest, showing similar conditions in the two experimental sessions, and after both interventions to characterize the results. The conventional statistical analysis (paired Student’s *t*-test) indicated the effect of IM_PA_ on performance, without modifications in physiological variables, at least by cause–effect analysis. The IM_PA_ protocol (2 sets of 15 repetitions applied 2 min before 30 s of tethered running) increased the absolute and relative mean running power (Table 1).

**Figure 2:**
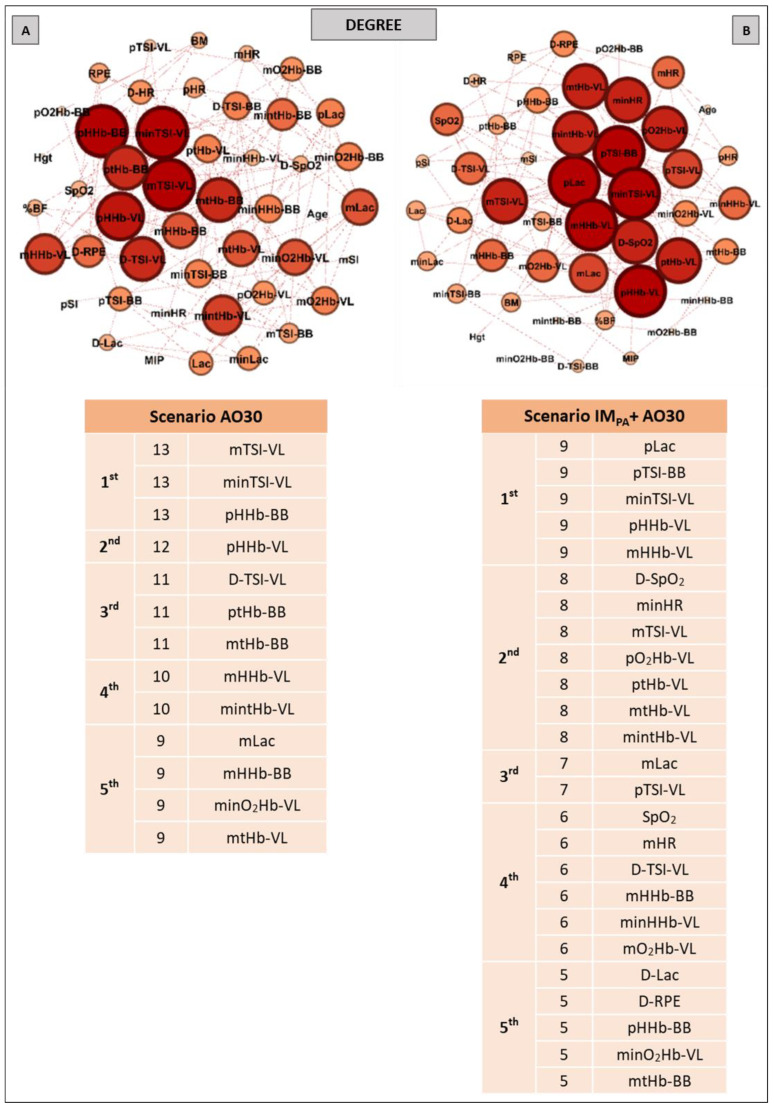
Graphs and top five node results obtained by complex network analysis using the Degree metric in two exercise scenarios: without inspiratory muscle pre-activation (Scenario AO30 shown in **Panel A**) and with inspiratory muscle pre-activation (Scenario IM_PA_ + AO30, **Panel B**). Graph visualizations were done using Gephi software (version 0.9.2, Paris, France). **Legend:** AO30 = all-out 30 s, IM_PA_ = inspiratory muscle pre-activation, Hgt = height, BM = body mass, %BF = percentage of body fat, MIP = maximal inspiratory pressure, SI = global strength index of inspiratory muscles, Lac = blood lactate, HR = heart rate, SpO_2_ = arterial oxygen saturation, RPE = perceived exertion, O_2_Hb = oxyhemoglobin relative concentration, HHb = deoxyhemoglobin relative concentration, tHb = total hemoglobin, TSI = tissue saturation index. Abbreviations accompanied by *p*, m, min and D represent peak, mean, minimum and delta (between final and initial results), respectively. Abbreviations accompanied by BB and VL represent the results in the biceps brachii and vastus lateralis, respectively.

**Figure 3:**
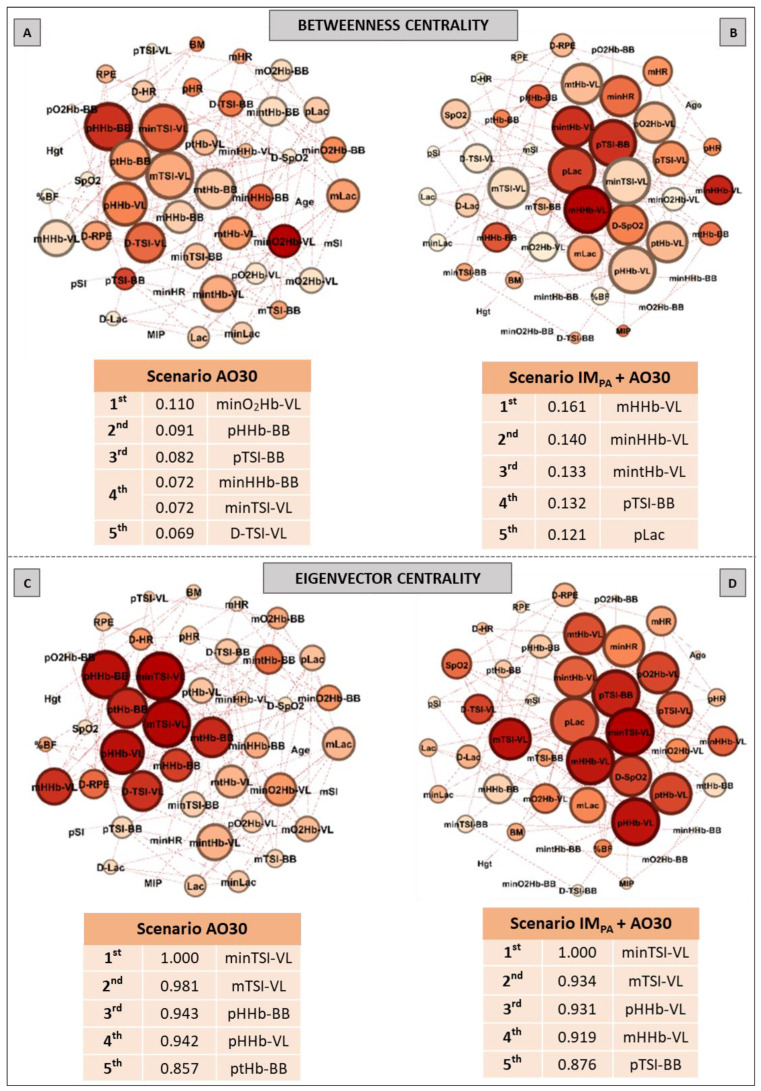
Graphs and top five node results obtained by complex network analysis using Betweenness Centrality (**Panels A,B**) and Eigenvector Centrality metrics (**Panels C,D**) in two exercise scenarios: without inspiratory muscle pre-activation (Scenario AO30, **Panels A,C**) and with inspiratory muscle pre-activation (Scenario IM_PA_ + AO30, **Panels B,D**). Graph visualizations were done using Gephi software (version 0.9.2, Paris, France). **Legend:** AO30 = all-out 30 s, IM_PA_ = inspiratory muscle pre-activation, Hgt = height, BM = body mass, %BF = percentage of body fat, MIP = maximal inspiratory pressure, SI = global strength index of inspiratory muscles, Lac = blood lactate, HR = heart rate, SpO_2_ = arterial oxygen saturation, RPE = perceived exertion, O_2_Hb = oxyhemoglobin relative concentration, HHb = deoxyhemoglobin relative concentration, tHb = total hemoglobin, TSI = tissue saturation index. Abbreviations accompanied by *p*, m, min and D represent peak, mean, minimum and delta (between the final and initial results), respectively. Abbreviations accompanied by BB and VL represent the results in the biceps brachii and vastus lateralis, respectively.

**Figure 4:**
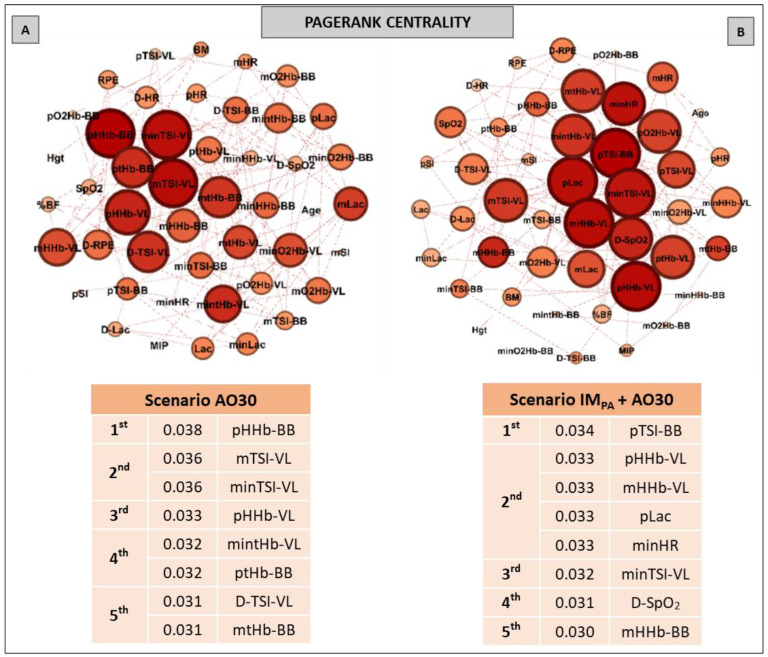
Graphs and top five node results obtained by complex network analysis using the Pagerank metric in exercise scenarios without inspiratory muscle pre-activation (Scenario AO30, at **Panel A**) and with inspiratory muscle pre-activation (Scenario IM_PA_ + AO30, at **Panel B**). Graph visualizations were done using Gephi software (version 0.9.2, Paris, France). **Legend:** AO30 = all-out 30 s, IM_PA_ = inspiratory muscle pre-activation, Hgt = height, BM = body mass, %BF = percentual of body fat, MIP = maximal inspiratory pressure, SI = global strength index of inspiratory muscles, Lac = blood lactate, HR = heart rate, SpO_2_ = arterial oxygen saturation, RPE = perceived exertion, O_2_Hb = oxyhemoglobin relative concentration, HHb = deoxyhemoglobin relative concentration, tHb = total hemoglobin, TSI = tissue saturation index. Abbreviations accompanied by *p*, m, min and D represent peak, mean, minimum and delta (between the final and initial results), respectively. Abbreviations accompanied by BB and VL represent the results in the biceps brachii and vastus lateralis, respectively.

Aiming to investigate the effect of IM_PA_ on node influences in an integrative analysis, we used centrality metrics (Degree, Betweenness, Eigenvector and Pagerank). In this way, Figure 2, Figure 3 and Figure 4 show the graphs and tables containing the top five ranked nodes returned from the complex network metrics in two exercise scenarios (without or with IM_PA_), considering the main variables in the interactions according to the metrics. When the metric values returned similar results for different nodes, we included these nodes in the top five list with the same importance classification. As informed in the methods section, 46 nodes were used to construct all the graphs in two exercise scenarios. After the complex network analysis, 147 edges and 119 edges were observed in the scenarios with and without IM_PA_ efforts, respectively (Figure 2, Figure 3 and Figure 4).

The results of the Degree metric in an exercise context are expressed in Figure 2. Thirteen nodes were highlighted in the first scenario (without IM_PA_); a high number of connections (13 links for the first nodes) and the important participation of muscle oxygenation responses for both BB and VL were visualized (Panel A). In the exercise preceded by IM_PA_ (Panel B), 25 nodes assumed the top five ranking, with special attention given to the peak blood lactate and muscle oxygenation responses in VL. In this scenario, HR and SpO_2_ also appear on the top five list.

Figure 3 shows the results of the Betweenness centrality (Panels A, B) and Eigenvector metrics (Panels C, D) obtained in the AO30 exercise, without or with IM_PA_ application. For the Betweenness, in the exercise without IM_PA_ (Panel A), minimum oxyhemoglobin in VL and many parameters in BB assumed the main positions. When IM_PA_ was applied (Panel B), the main returned nodes were related to muscle oxygenation in VL, and the peak blood lactate once again appeared in the top five ranking. For the Eigenvector metric, only muscle oxygenation responses in both muscles configured the top five nodes in the exercise without the IM_PA_ scenario (Panel C). IM_PA_ increased the values of this metric and modified the main players (Panel D). In this last case, TSI and HHb in VL assumed the first node positions.

Pagerank was the last centrality metric adopted for the investigation of the IM_PA_ influence on exercise by network analysis (Figure 4). According to this metric, the top five nodes in the non-IM_PA_ (Panel A) exercise scenario were composed of muscle oxygenation responses in VL and BB. After IM_PA_, the exercise scenario was modified (Panel B), and pLac and minHR figured within the main nodes for the Pagerank—both in second place.

**Figure 5:**
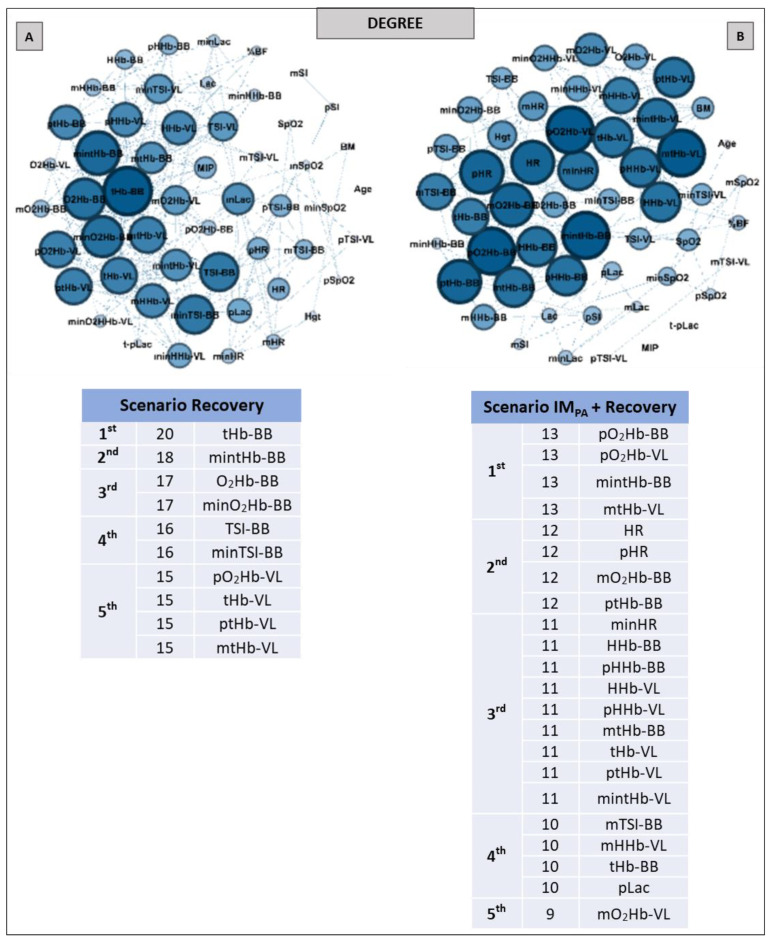
Graphs and top five node results obtained by complex network analysis using the Degree metric in two recovery scenarios: without inspiratory muscle pre-activation (Scenario Recovery, at **Panel A**) and with inspiratory muscle pre-activation (Scenario IM_PA_ + Recovery, at **Panel B**). Graph visualizations were done using Gephi software (version 0.9.2, Paris, France). **Legend:** AO30 = all-out 30 s, IM_PA_ = inspiratory muscle pre-activation, Hgt = height, BM = body mass, %BF = percent body fat, MIP = maximal inspiratory pressure, SI = global strength index of inspiratory muscles, Lac = blood lactate, HR = heart rate, SpO_2_ = arterial oxygen saturation, O_2_Hb = oxyhemoglobin relative concentration, HHb = deoxyhemoglobin relative concentration, tHb = total hemoglobin, TSI = tissue saturation index. Abbreviations accompanied by *p*, m and min represent peak, mean and minimum values, respectively. Abbreviations accompanied by BB and VL represent the results in the biceps brachii and vastus lateralis, respectively.

**Figure 6:**
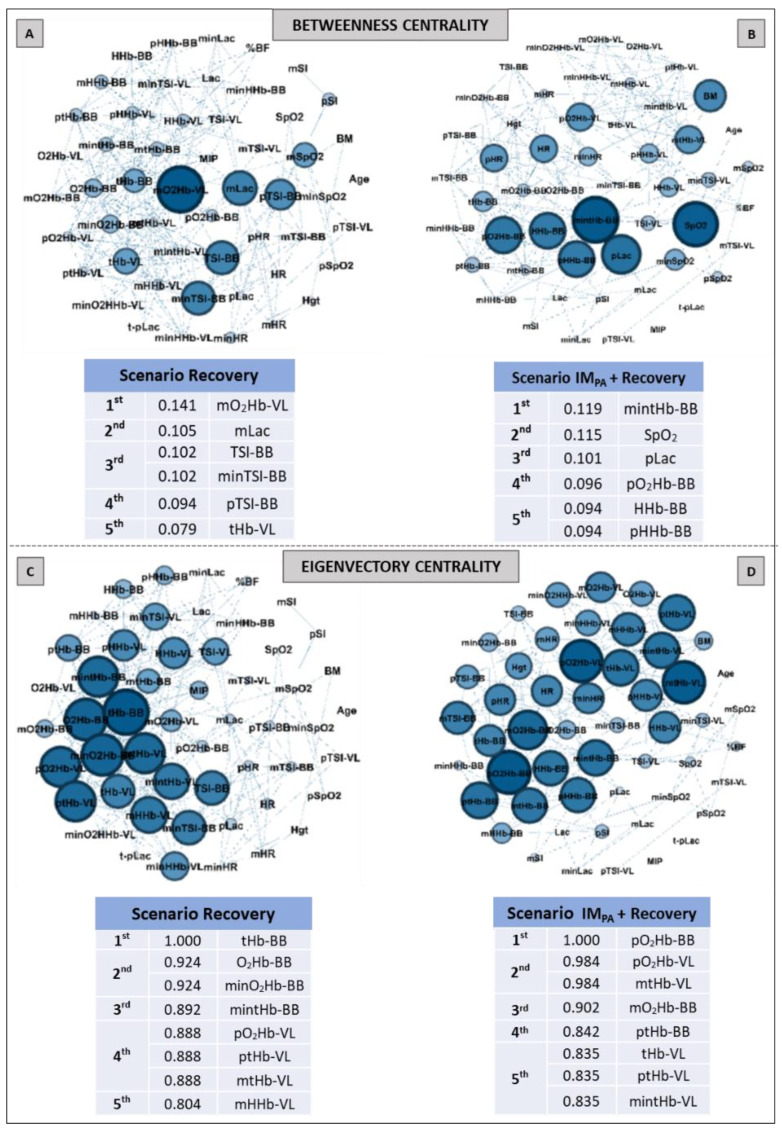
Graphs and top five node results obtained by complex network analysis using Betweenness Centrality (**Panels A,B**) and Eigenvector Centrality (**Panels C,D**) metrics in two recovery scenarios: without inspiratory muscle pre-activation (Scenario Recovery, at **Panel A,C**) and with inspiratory muscle pre-activation (Scenario IM_PA_ + Recovery, at **Panels B,D**). Graph visualizations were done using Gephi software (version 0.9.2, Paris, France). **Legend:** AO30 = all-out 30 s, IM_PA_ = inspiratory muscle pre-activation, Hgt = height, BM = body mass, %BF = percentage of body fat, MIP = maximal inspiratory pressure, SI = global strength index of inspiratory muscles, Lac = blood lactate, HR = heart rate, SpO_2_ = arterial oxygen saturation, O_2_Hb = oxyhemoglobin relative concentration, HHb = deoxyhemoglobin relative concentration, tHb = total hemoglobin, TSI = tissue saturation index. Abbreviations accompanied by *p*, m and min represent peak, mean and minimum values, respectively. Abbreviations accompanied by BB and VL represent the results in the biceps brachii and vastus lateralis, respectively.

**Figure 7:**
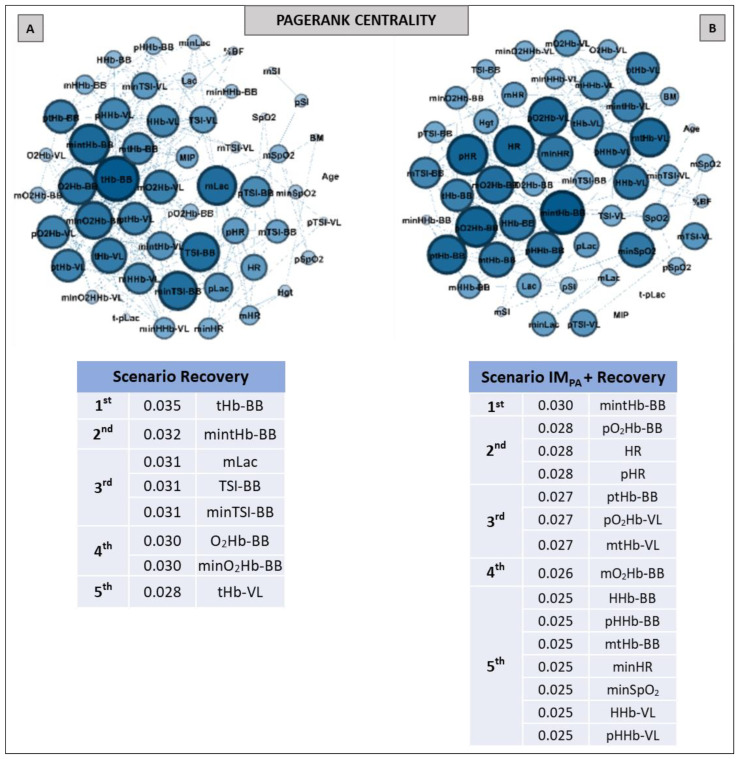
Graphs and top five node results obtained by complex network analysis using the Pagerank metric in two recovery scenarios: without inspiratory muscle pre-activation (Scenario Recovery, at **Panel A**) and with inspiratory muscle pre-activation (Scenario IM_PA_ + Recovery, at **Panel B**). Graph visualizations were done using Gephi software (version 0.9.2, Paris, France). **Legend:** AO30 = all-out 30 s, IM_PA_ = inspiratory muscle pre-activation, Hgt = height, BM = body mass, %BF = percentage of body fat, MIP = maximal inspiratory pressure, SI = global strength index of inspiratory muscles, Lac = blood lactate, HR = heart rate, SpO_2_ = arterial oxygen saturation, O_2_Hb = oxyhemoglobin relative concentration, HHb = deoxyhemoglobin relative concentration, tHb = total hemoglobin, TSI = tissue saturation index. Abbreviations accompanied by *p*, m and min represent peak, mean and minimum values, respectively. Abbreviations accompanied by BB and VL represent the results in the biceps brachii and vastus lateralis, respectively.

In the same way as that presented by the exercise scenarios, we studied the recovery after efforts conducted without or with IM_PA_ (Figure 5, Figure 6 and Figure 7). Using 52 nodes, the complex network model returned 231 and 192 undirected edges for the first and second conditions, respectively.

With regard to the Degree metrics, all of the nodes that appeared as top five in the recovery exercise scenario without IM_PA_ were associated with muscle responses, especially in BB, considering the less active muscle in the AO30 running exercise (Figure 5, Panel A). For example, the most significant node in this scenario was the total hemoglobin in BB, with 20 connections. In contrast, in the IM_PA_ + recovery scenario, we observed the increase in the number of nodes completing the top five list (10 versus 22 nodes, in Panels A and B, respectively), with HR and pLac figuring as important roles in this centrality metric.

The Betweenness and Eigenvector results are expressed in Figure 6 (Panels A–D). Muscle oxygenation (in BB and VL) and mean blood lactate were set in the top five in the recovery scenario (Figure 6, Panel A). When IM_PA_ was applied, there was a modification in the main nodes for this metric. SpO_2_ and pLac assumed the second and third most important places in this scenario, and only BB muscle responses were highlighted (Figure 6, Panel B). For the Eigenvector metric, only oxyhemoglobin, deoxyhemoglobin and total hemoglobin in BB and VL characterized the top five ranking for both scenarios, showing similar metric values and a similar number of nodes in two cases.

The graphs and table with the main nodes observed by the Pagerank metric in the recovery scenarios are shown in Figure 7. The differences observed for this metric in two recovery scenarios were the higher number of nodes and the appearance of the physiological measurements (HR, SpO_2_ and Lac) in the top five ranking in Panel B compared to Panel A (i.e., in the recovery after the exercise preceded by IM_PA_).

## 4. Discussion

This study aimed to understand the effects of IM_PA_ on high-intensity running power and recovery applied to active subjects. So, in a novel and innovative way of investigating the impacts of IM_PA_ on high-intensity running and recovery, we proposed the identification of the interaction among physical characteristics, physiological responses and muscle oxygenation in more and less active muscle using a complex network model. The data analysis confirmed our hypotheses. The IM_PA_ at 40% of the MIP improves the running power and provokes changes in the graph’s configuration in the exercise and recovery. The centrality metrics obtained from a complex network model were capable of detecting the impact of this pre-activation strategy on physiological interactions. In an additional and interesting manner, this computational model detected the connections and adjustments expected by theoretical and practical explanations about IM_PA_ on an organism.

Our main objective was not to analyze the equality or difference between physiological responses in high-intensity effort and recovery preceded or not preceded by IM_PA_. We consider the complexity of the physiological responses to the demands imposed by exercise and return to rest, and this does not occur in isolation. We resort to a multidimensional analysis proposed by complex networks, based on highly robust measures and using accurate wearable technologies. Our interest was to verify which adjustments actually occur in these different scenarios (without and with IM_PA_ before exercise and also in recovery after these interventions in a running context) using a more integrated approach, associating different physiological parameters interpreted as nodes of the networks. In this sense, as observed in a previous study in Judo (Cirino et al., 2021), IM_PA_ seems to prepare the body for more physical work, at least through a more holistic analysis.

The option to investigate the effects of IM_PA_ on high-intensity running exercise is justified by the importance of these muscles in the performance context [1], as well as by previous studies indicating that the warm-up of this musculature is able to enhance the subsequent physical effort [18,19]. Here, by analyzing equalities and differences using a paired t-test, we already observed a higher absolute and relative peak and mean power to the trial accomplished after respiratory intervention. These results corroborate those of other studies conducted with athletes [18,19,20,66], which also observed a boost in performance after the warm-up of IMs. Note that the mechanical inspiratory strategy adopted here was characterized by 2 sets of 15 breaths, with 40% of the MIP using the POWERbreathe IM trainer (K5 model). While maintaining the flow restriction (40% of the individual MIP) employed in other investigations [16,19,66], we used fewer than 30 breath repetitions per set. This option occurred due to our sample being composed of active subjects without previous muscle inspiratory training. Based on our findings, and corroborating those of Cirino et al. [21], the IM_PA_ (2 × 15 breaths at 40% of the MIP) promoted positive effects on performance, with running accomplished in an ergometer constructed to record power signals with a high-capture frequency. We emphasize that, although under laboratory conditions, this tethered test is very similar to high-intensity running conducted in the field [42].

Regarding the integrated analysis, multidimensional approaches have been strongly recommended to understand non-isolated biological phenomena [46,47,48,49,50,51]. Recently, Verschueren et al. [67] conducted an interesting systematic review about the fatigue process, discussing the importance of a multidimensional approach. They appointed the complex network model as a possible method. Likewise, Balagué et al. [68] suggest network exercise physiology with a focus on basic laws of interactions among diverse physiological systems. We believe that fatigue and exercise response depend on many players, and the effects of IM_PA_ also follow this path. According to Perry [69], NIRS-derived muscle parameters should be implemented in a manner comparable to routinely used conventional internal parameters (e.g., HR, lactate, VO_2_, RPE), and the NIRS data require computational tools to explain the observational intra- and inter-individual variability responses to exercise. In this sense, based on accurate peripheral measurements obtained by the NIRS technique in two different muscles, as well as on the monitoring of some systemic physiological responses (HR, SpO_2_ and blood lactate), we chose the complex network approach to interpret our data. To the best of our knowledge, the network centrality metric has not yet been applied to IM investigations associated with NIRS measurements, but it can certainly contribute to the integrated and multidimensional understanding of IM_PA_ effects on exercise and recovery, as will be discussed.

Although there are many measures of centrality [70], we chose four of them for the current investigation (Degree, Betweenness, Eigenvector and Pagerank centralities). Our option was based on previous studies involving exercise physiology [21,40,41,42,43] and a recent paper that discussed the consistency and differences between centrality measures across distinct classes of networks [52].

The simplest and most popular measure of centrality is the node degree centrality, which indicates the number of connections of this node on the graph [71]. So, this is first shown in our network results (Figure 2 and Figure 5, for exercise and recovery scenarios, respectively). Betweenness centrality expresses the importance of elements (vertex/edge) involved in a network, evaluating the traffic in communication networks, and also identifies critical intersections in a road network [53,72]. These metric results were expressed in Figure 3 and Figure 6 (Panels A and B). On the other hand, Eigenvector centrality is a measure related to prestige. The prestige of node *i* is related to the prestige of their neighbor’s (assuming that centrality of node *i* is proportional to the sum of centrality of node *i*´s neighbors) [53,54]. Our eigenvector results are shown in Figure 3 and Figure 6 (Panels C and D). Finally, Pagerank is the most recent algorithm compared to the other three and defines a link analysis method to evaluate a user´s influence, so not only is the immediate information flow incorporated but the information flow after that is also considered [73,74]. Its importance is not necessarily established by the number of node connections but rather by which other nodes are connected to it. The pagerank values obtained in our study are shown in Figure 4 and Figure 7 for exercise and recovery scenarios, respectively.

## 5. Centrality Metrics in Exercise Scenarios

The main findings involving complex networks and exercise scenarios revealed that IM_PA_ reduced the number of connections between each node (degree value) but substantially increased the number of main nodes within the networks (top five ranking), which can be viewed by the use of the Degree metric (Figure 2, Panels A and B). The number of important nodes related to muscle oxygenation measures was greater for the locomotor muscle in the running effort (vastus lateralis) when IM_PA_ was performed. Still, in the scenario with IM_PA_, systemic physiological responses appeared as very important nodes in the network. For example, the pLac, which was not highlighted in the first scenario, attained the first place in the ranking of the degree together with the deoxyhemoglobin in the VL and the TSI in the BB. SpO_2_ and HR also only appeared prominently in the network constructed in the IM_PA_ + AO30 scenario. During high-intensity running with similar times of the exercise as those of the present study, the VL muscle is activated to maintain exercise demands [75], which may justify the large number of studies focused on investigating changes in the oxygenation of this muscle, as pointed out by Perrey & Ferrari, 2018 [29]. Findings related to the Degree metric confirm the importance of VL in an integrative way, as investigated in a high-intensity running context. Furthermore, these results are very interesting, signaling an impact of IM_PA_ in increasing the importance of the most active muscle in running, as observed by complex analysis (VL-related nodes), which is possibly mediated by more systemic connections.

The results for the pathway metric (Betweenness centrality) also signal an increase in the importance of VL oxygenation within the network when IM_PA_ was applied (see Figure 3, Panels A and B). Again, blood lactate appears in the top five ranking (Figure 3, Panel B), with the metric value being higher than that placed first in the scenario of AO30 without pre-activation (0.110 observed by minimum deoxyhemoglobin in VL vs. 0.121 to the peak of blood lactate). The list of the main prestigious nodes was implemented with VL responses after IM_PA_, with four of the five main eigenvector values being related to oxygenation in this locomotor muscle (highlighting the TSI and deoxyhemoglobin values in VL). Finally, the Pagerank analysis corroborates the significant importance of systemic responses (pLac, minHR and D-SpO_2_) when IM_PA_ was adopted (Figure 4, Panel B). Considering that a high Pagerank value assumes that a node acts critically when it is linked to others that perform essential functions in the probability distributions to each node [42,74], deoxygenation in the less active muscle (BB) proved to be a very representative node in the scenario without IM_PA_. On the other hand, not only for Pagerank but also for the other metrics, the blood lactate concentration is actually revealed as a “chief messenger” [76] or a “fulcrum” (as recently named by Brooks [26]) in the complex network model as well. In our case, its prominence was highly enhanced by IM_PA,_ which was observed by Cirino at al. [21] using IM_PA_15 and 40% of the MIP before a Judo match.

We emphasize that muscle oxygenation nodes were highlighted in all graphs plotted for high-intensity scenarios. This suggests that relative concentrations of oxy-, deoxy- and total hemoglobin detected by NIRS in one or more muscles may, in fact, deepen the understanding of exercise effects, as indicated by other authors [29,34,77]. In a muscle oxygenation context, special attention has been dedicated to deoxyhemoglobin (HHb), which represents the utilization of O_2_ by peripheral tissue and is potentially unaffected by changes in blood volume and arterial hemoglobin concentration in high-intensity exercise [78,79]. For example, recently, Yogev and colleagues [80] compared the respiratory compensation point with muscle oxygen saturation in locomotor and non-locomotor muscles using the HHb breakpoint. Here, the complex network model confirmed their significance in an integrative analysis, with HHb nodes attaining the top three in all scenarios and metrics. Likewise, as appointed by Richard & Billaut [39], muscle oxygenation analysis can also improve the comprehension about the effects of IM warm-up on exercise.

Taken together, the results of the multidimensional analysis adopted for exercise scenarios suggest that, in fact, IM_PA_ promotes positive changes in the interaction of peripherical and systemic responses. This interaction emphasizes nodes related to the locomotor running muscle, as well as the pLac and HR nodes.

## 6. Centrality Metric in Recovery Scenarios

After high-intensity exercise is interrupted in the AO30 applied here, some responses quickly return to rest values, and other adjustments occur later [34,81]. In addition to contributing to better interactions in physical effort, we expected that IM_PA_ could also bring about positive changes in post-effort, perhaps detected by network analysis. This hypothesis was also confirmed. Unlike what happens with effort when the most active muscle needs to receive more blood flow during the task, the less active muscles can contribute significantly to the recovery process—assisting in the lactate shuttle, for example. Here, the importance of the less active muscle was detected by a multidimensional approach, with many respiratory responses in the BB acting with the main nodes in different metrics.

Following the exercise scenario, IM_PA_ likewise increased (more than twice) the number of highlighted nodes in the degree centrality (Figure 5, Panels A and B). Additionally, this strategy contributed to the HR and pLac nodes attaining top five rankings, suggesting that the prior respiratory series at 40% MIP can also contribute to post-effort. Tong and Fu [22] had already observed the impacts of acute inspiratory load in the recovery process, but they used other parameters and a conventional statistical analysis. Moreover, recovery benefits have been observed after the application of the IM training, confirming the relationship between the efficiency of respiratory muscles and post-exercise recovery [2,5]. Here, by the simplest and most popular measure of centrality, the impacts of IM_PA_ were confirmed.

Regarding the Betweenness centrality, the muscle oxygenation nodes in the recovery scenario (Figure 6, Panel A) were prominent, with special attention to the O_2_ delivery nodes in less active muscle. However, IM_PA_ made sure that only the BB responses were present in the top five ranking, which were mediated by the SpO_2_ and pLac nodes (second and third positions, respectively) in this pathway metric (Figure 6, Panel B). Corroborating investigations that used other approaches [34,35,37], the less active muscle stood out in the recovery process according to the Betweenness metric. So, the effectiveness of IM_PA_ in generating this highlight was also confirmed by the network model.

The analysis of prestigious nodes (Eigenvector centrality) only highlighted nodes related to muscle breathing in both recovery scenarios (Figure 6, Panels C and D). In this case, the BB total hemoglobin and oxyhemoglobin in this same muscle showed the highest Eigenvector value in recovery without and with IM_PA_, respectively. As this metric is related to the prestige of the neighboring nodes [55,56], the graph analysis again confirmed the importance of muscle O_2_ delivery in the recovery process. Here, the Pagerank metric indicated the mean blood lactate as one the top five nodes in the recovery scenario without IM intervention (Figure 7, Panel A). In the present study, this was the first time that blood lactate was ranked in a scenario without IM_PA_. This metabolite being highlighted by a centrality metric such as Pagerank further confirms its importance as a pathway, even without a prior stimulus such as IM_PA_.

Finally, we emphasize that, in all the graphs shown in our study (exercise and recovery, with or without IM_PA_ scenarios), the physical characteristics such as age, height, body mass and fat percentage, as well as MIP and SI, were not highlighted by any metric. This was different from the other studies with complex networks and exercise developed by our group, in which physical characteristics were quite prominent [21,40,41,42]. However, physical measurements were more related to experimental designs and mainly aim on those occasions (i.e., power and jumps in basketball, running to exhaustion in elite athletes and a Judo match). Based on theoretical and practical assumptions that support the previous stimulus to IMs, we expected that nodes related to muscle oxygenation and systemic responses would show a greater prominence. Even at this point, the centrality metrics have been shown by a complex network model to be robust and sensitive to different experimental models.

The limitation of the multidimensional approaches adopted here is in the selection of input parameters as network nodes. Despite this, our design sought to select the best measures within our reach and at the frontier of knowledge relevant to our objectives. In this sense, wearable technologies that do not hinder with the execution of IM_PA_, exercise and recovery and the high uptake of physiological signals were adopted to generate the nodes.

In summary, the IM_PA_ protocol (load at 40% of the MIP) was effective at increasing the running power in high-intensity tethered efforts, and the complex network model contributed to improving the interpretation into physiological adjustments related to the impact of IM_PA_ on exercise and recovery. Centrality metrics highlighted the nodes related to muscle oxygenation responses (in more and less active muscles) as significant to all scenarios, and systemic physiological responses mediated this impact, especially after IM_PA_ application. Thus, in practical terms, we recommend this respiratory strategy to improve exercise and recovery, and we further support a multidimensional approach using complex network modelling to understand the physiological adjustments to these and other conditions.

## Figures and Tables

**Table 1:** Peak, mean and minimum values of the absolute running power (W) and the power relative to body mass (W·kg^−1^) during the AO30 exercise (*n* = 15) and AO30 with inspiratory muscle pre-activation (IM_PA_ + AO30), and physiological responses at rest and after effort in these two experimental sessions.

	AO30	IM_PA_ + AO30	*p*
Mechanical responses			
pP (W)	2296.7 ± 126.3	2501.1 ± 115.6	0.05
mP (W)	1706.9 ± 104.7	1894.0 ± 93.8	0.02
minP (W)	1210.3 ± 89.2	1372.3 ± 81.8	0.06
pP (W·kg^−1^)	31.4 ± 1.5	34.0 ± 1.4	0.04
mP (W·kg^−1^)	23.3 ± 1.1	25.8 ± 1.0	0.02
minP (W·kg^−1^)	16.5 ± 1.0	18.6 ± 07	0.06
FI (%)	47.2 ± 2.4	44.7 ± 2.6	0.32
Physiological responses			
at rest			
LAC (mM)	0.8 ± 0.1	0.8 ± 0.1	0.88
HR (bpm)	65 ± 3	66 ± 3	0.75
SPO_2_ (%)	97.4 ± 0.2	97.3 ± 0.2	0.50
TSI_BB (%)	66.1 ± 0.9	68.3 ± 0.7	0.07
TSI_VL (%)	73.7 ± 0.8	72.8 ± 0.8	0.27
Physiological responses			
after effort			
LAC peak (mM)	17.2 ± 0.6	16.2 ± 0.6	0.07
time to pLAC	8.1 ± 0.6	8.4 ± 0.6	0.76
HR (bpm)	176 ± 2	179 ± 2	0.11
SPO_2_ (%)	96.9 ± 0.3	96.9 ± 0.3	0.87
TSI_BB (%)	32.2 ± 1.8	34.1 ± 1.2	0.38
TSI_VL (%)	50.1 ± 1.7	49.8 ± 1.6	0.78

Results are expressed in mean ± SEM. pP = peak power, mP = mean power, minP = minimum power, FI = fatigue index, LAC = blood lactate, HR = heart rate, SPO_2_ = arterial oxygen saturation, TSI = tissue saturation index, BB = biceps brachii, VL = vastus lateralis. (*p* ≤ 0.05).

## Data Availability

All data are stored in the database of the Laboratory of Applied Sports Physiology—FCA—UNICAMP. The data will be made available upon request.

## Supplementary Materials

The following supporting information can be downloaded at: https://www.mdpi.com/article/10.3390/biology11070963/s1. File S1: Complex network centrality metrics: Degree, Betweenness, Eigenvector, and Pagerank. References [82,83,84,85,86] are cited in the supplementary materials.
