# Supplementary material for: Complex Network Model Reveals the Impact of Inspiratory Muscle Pre-Activation on Interactions among Physiological Responses and Muscle Oxygenation during Running and Passive Recovery
Source: Biology (Basel). 2022 Jun 25;11(7):963. doi: 10.3390/biology11070963 (PMC9311794; doi:10.3390/biology11070963)
Supplement: Supplementary file 1 [file biology-11-00963-s001.zip › biology-1765386-supplementary.pdf]

Complex Network Model Reveals the Impact of Inspiratory Muscle Pre-Activation on Interactions among Physiological Responses and Muscle Oxygenation during Running and Passive Recovery

Fúlvia Barros Manchado-Gobatto[1,*]; Ricardo Silva Torres[2]; Ainta Brum Marostegan[1]; Felipe Marroni Rasteiro[1]; Charlini Simoni Hartz[3]; Marlene Aparecida Moreno[3]; Allan Pinto[4,5]; and Claudio Alexandre Gobatto[1]

[1]*Laboratory of Applied Sport Physiology, School of Applied Sciences, University of Campinas, Limeira, Sao Paulo, Brazil;*[1]

[2]*Department of ICT and Natural Sciences, Norwegian University of Science and Technology*

[3]*Postgraduate Program in Human Movement Sciences, Methodist University of PiracicabaPostgraduate Program in Human Movement Sciences, Methodist University of Piracicaba, Piracicaba, São Paulo, Brazil.*

[4]*Department of Sport Sciences, Faculty of Physical Education, University of Campinas, 13083-851, Campinas, São Paulo, Brazil.*

[5]*Brazilian Synchrotron Light Laboratory (LNLS), Brazilian Center for Research in Energy and Materials (CNPEM), 13083-970, Campinas, Sao Paulo, Brazil.*

[*]***Corresponding author:***
Fúlvia B. Manchado-Gobatto
fgobatto@unicamp.br

Supplemental File S1: Complex network centrality metrics: Degree, Betweenness, Eigenvector, and Pagerank.

Centrality measures have been widely used in network analyses to estimate the importance of nodes that compose a graph [5]. There are several formulations of centrality in the literature, and the decision of which one should be adopted depends on the target problem. For instance, in social networks, the notion of centrality or importance could be formulated as a degree of vertices since a high-degree may characterize a popular or a prestigious node or person. Conversely, in communication networks, the centrality of a node could be formulated as the number of shortest paths that include that vertex, since shortest paths are preferable to processing and forwarding messages. This appendix provides an overview of the centrality measures considered in this work.

An undirected graph $G = (\mathcal{V}, \mathcal{E})$ is a structure that consists of a finite set of vertices $\mathcal{V}$ and a finite set of edges $\mathcal{E}$. The number of vertices in a graph G is given by the cardinality of the set of vertices, i.e., $N \equiv |\mathcal{V}|$, while the number of edges comprises the cardinality of the set $\mathcal{E}$. The vertices are also referred to as nodes or points of the graph and they are identified by labels. The edges (also called links or lines) are defined as $E \subseteq (i, j)|(i, j) \in V^2 \wedge x \neq y$ and represent the linkage between pairs of nodes. An edge is said to be incident in nodes i and j and two nodes joined by an edge are referred to as adjacent or neighbouring [2, 4]. In a direct graph, the edges that comprise the graph carry on the notion of orientation. Thus, the edge between the nodes i and j are denoted by the ordered pair $(i, j) \neq (j, i)$ and we say that the edge is ongoing in j and outgoing from i. There is also the situation in which the edges have a numerical value that quantifies the intensity of the connection between two nodes. The graphs that follow this representation is refereed to as
weighted graphs and are defined as $G = (\mathcal{V}, \mathcal{E}, \mathcal{W})$, $\mathcal{W} \subseteq \mathbb{R}^+$.

An unweighted graph G can be represented by a $|V| \times |V|$ matrix A, named as the adjacency matrix of graph G. The elements of the matrix A are given by:

$$a_{ij} = \begin{cases} 1 & \text{if there is an edge from node } j \text{ to node } i \\ 0 & \text{otherwise} \end{cases} \tag{S1}$$

The adjacency matrix of a weighted graph can also store the weights of the edges. In this case, a special value (e.g., a negative or a large value) may be used to represent missing edges. Figure S1 presents an example of a weighted and undirected graph, which we will use to exemplify the computation of the centrality metrics presented in this appendix.

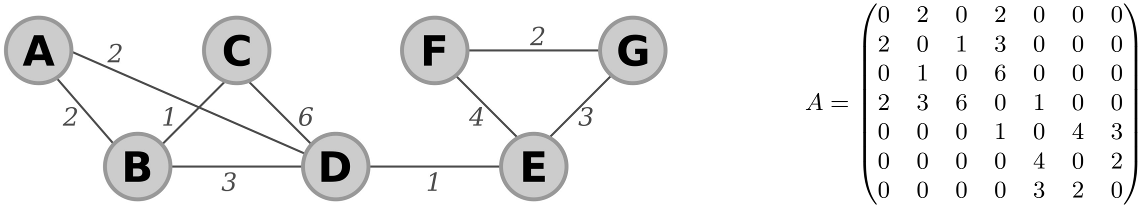

(a) Graph G.

$$A = \begin{pmatrix} 0 & 2 & 0 & 2 & 0 & 0 & 0 \\ 2 & 0 & 1 & 3 & 0 & 0 & 0 \\ 0 & 1 & 0 & 6 & 0 & 0 & 0 \\ 2 & 3 & 6 & 0 & 1 & 0 & 0 \\ 0 & 0 & 0 & 1 & 0 & 4 & 3 \\ 0 & 0 & 0 & 0 & 4 & 0 & 2 \\ 0 & 0 & 0 & 0 & 3 & 2 & 0 \end{pmatrix}$$

(b) Adjacency matrix.

Figure S1: Example of an undirected and weighted graph G with $V = \{A, B, C, D, E, F, G\}$ and $E = \{(A, B) \mapsto 2, (A, D) \mapsto 2, (B, C) \mapsto 1, (B, D) \mapsto 3, (C, D) \mapsto 6, (D, E) \mapsto 1, (E, F) \mapsto 4, (E, G) \mapsto 3, (F, G) \mapsto 2\}$.

Degree centrality

The number of neighbors of nodes provides meaningful information in terms of their importance. A node with a high degree can be interpreted as an essential source of information or a node with a high influence capacity [2]. Considering an undirected and weighted graph G, the degree centrality can be defined as:

$$c_i^D = \sum_{j=1}^{N} \phi_{ij}, \quad \phi_{ij} = \begin{cases} 1 & a_{ij} > 0 \\ 0 & \text{otherwise} \end{cases}, \quad i = 1, 2, \cdots, N. \tag{S2}$$

where c_i is the degree of node i and N is the number of nodes. The normalized degree centrality is defined as:

$$nc_i^D = \frac{c_i^D}{N-1} \tag{S3}$$

Tables S1 and S2 show the absolute and the normalized degree centrality for each node of the graph presented in Figure S1.

Table S1: Degree centrality for the vertices in G.

c_A^D	c_B^D	c_C^D	c_D^D	c_E^D	c_F^D	c_G^D
2	3	2	4	3	2	2

Table S2: Normalized degree centrality for the vertices in G.

nc_A^D	nc_B^D	nc_C^D	nc_D^D	nc_E^D	nc_F^D	nc_G^D
0.333	0.500	0.333	0.667	0.500	0.333	0.333

Eigenvector centrality

Although the degree centrality measure provides meaningful information in terms of nodes importance, there are some drawbacks with this measure that could prevent its proper use in some applications. Suppose that node A is connected with other nodes that are themselves important, and also another node D is connected to the same amount of nodes but with less importance. In this case, the degree centrality measure produces equal scores for both nodes A and D, which might not be appropriate in some applications. The eigenvector centrality tries to overcome this limitation by producing a centrality score that is proportional to the sum of the scores of neighbors.

$$\begin{gathered} c_i = \textstyle\sum_{j=1}^{N} a_{ij} c_j, \quad i = 1, 2, \cdots, N. \\ \mathbf{c} = \mathbf{A}\mathbf{c} \quad \equiv \quad (\mathbf{A} - \mathbf{I})\mathbf{c} = 0 \end{gathered} \tag{S4}$$

where vector $\mathbf{c}$ is the unknown centrality score, $\mathbf{A}$ is the adjacency matrix and N is the number of nodes. This formulation leads to a homogeneous system which admits the trivial solution, i.e., $\mathbf{c} = 0$ or non-trivial solutions if $det(\mathbf{A} - \mathbf{I}) = 0$, which might appear in few cases. The standard procedure to solve this problem is to multiply the left side of this equation by a constant λ, as shown in Equation (S5):

$$\begin{gathered} \lambda c_i^E = \textstyle\sum_{i}^{N} a_{ij} c_j^E, \quad i = 1, 2, \cdots, N. \\ \lambda \mathbf{c} = \mathbf{A}\mathbf{c} \end{gathered} \tag{S5}$$

where c_i^E is the eigenvector centrality of node i. Similarly, in the matrix notation, $\mathbf{c}$ is an N-dimensional vector whose entry i represents the centrality score of node i. This leads to the problem of finding the *eigenvalues* (λ) and the *eigenvectors* ($\mathbf{c}$) of the adjacency matrix $\mathbf{A}$. Finally, the eigenvector centrality of all nodes in graph G is the eigenvector associated with the dominant eigenvalue found for Equation (S5). There are several numerical algorithms that could be used to find the dominant eigenvector and its associate eigenvalue, such as the *power iteration* method [4]. This method starts with a non-zero vector $\mathbf{v}_0$, for instance, all-ones vector, and then iterates over the recurrent relation $\mathbf{v}_{k+1} = \frac{\mathbf{A}\cdot\mathbf{v}_k}{\|\mathbf{v}_k\|}$, until convergence. We can say that the method converges if $|\lambda_k - \lambda_{k+1}| < \epsilon$), where λ_k and λ_{k+1} are the associate eigenvalues of the eigenvectors $\mathbf{v}_k$ and $\mathbf{v}_{k+1}$, respectively. Table S3 illustrates the eigenvector centrality obtained with the power iteration method, considering the graph presented in Figure S1.

Pagerank centrality

Similarly to the eigenvector centrality, the Pagerank centrality also encodes the centrality of a node considering the centrality score of its neighbors. In the eigenvector centrality, the importance of a node is

Table S3: Computation of the Eigenvector centrality scores for graph G using the power iteration methods with an $\epsilon = 10^{-4}$.

Iteration	ϵ	c_A^E	c_B^E	c_C^E	c_D^E	c_E^E	c_F^E	c_G^E
0	0.00000	1.0000	1.0000	1.0000	1.0000	1.0000	1.0000	1.0000
1	0.14857	0.5000	0.7500	0.5000	1.0000	0.7500	0.5000	0.5000
2	0.03000	0.7000	0.8000	0.7000	1.0000	0.8000	0.5000	0.5000
3	0.01240	0.6000	0.8000	0.6000	1.0000	0.6667	0.4333	0.4333
4	0.00565	0.6750	0.8250	0.6750	1.0000	0.7000	0.4125	0.4125
5	0.00263	0.6348	0.8174	0.6348	1.0000	0.6348	0.3870	0.3870
6	0.00124	0.6677	0.8339	0.6677	1.0000	0.6518	0.3754	0.3754
7	0.00058	0.6501	0.8279	0.6501	1.0000	0.6206	0.3641	0.3641
8	0.00028	0.6650	0.8368	0.6650	1.0000	0.6288	0.3583	0.3583
9	0.00013	0.6570	0.8335	0.6570	1.0000	0.6140	0.3531	0.3531
10	0.00006	0.6639	0.8380	0.6639	1.0000	0.6178	0.3502	0.3502

proportional to the sum of the integral centrality score of its neighbors, which means that a high centrality node connected to many other nodes makes its neighbors have also a high centrality. In this context, a node became important whether it has high connectivity or whether it has few connections with very important neighbors [2]. This behavior is not desirable in the context that a node is not important just because it is connected to one important node. Conversely, the Pagerank centrality adopts a different strategy to spread importance: the centrality of a node is proportional to its neighbors' centrality divided by its out-degree [3]. This means that a node highly connected share a small portion of its centrality to all other ones connected to it, as defined in Equation (S6):

$$c_i^{PR} = (1-q)\sum_{j=0}^{N} a_{ij}\frac{c_j^{PR}}{k_j^{out}} + \frac{q}{N}, \quad i = 1, 2, \cdots, N. \tag{S6}$$

where c_i^{PR} is the Pagerank value for node i, k_j is the out-degree of node j, if such degree is positive, or $k_j = 1$ if the out-degree is zero. N is the number of nodes and q is a probability, or a damping factor, that controls the mix between random walk and random jump, which are two stochastic processes used for modeling an agent walking through the graph. These processes allows for measuring the probability of arriving at that node after a large number of iterations [3]. Thus, the Pagerank values represent the probability of reaching a node in the graph considering a random walk through the graph, starting from a random node. Finally, it is important to notice that this formulation can be extended also for an undirected graph. In this case, the random walk considers both directions of the edges.

Table S4 shows the Pagerank values for the graph introduced in Figure S1, considering the implementation of the Pagerank algorithm available in the Gephi software [1].

Table S4: Pagerank centrality for the vertices in G.

c_A^{PR}	c_B^{PR}	c_C^{PR}	c_D^{PR}	c_E^{PR}	c_F^{PR}	c_G^{PR}
0.11	0.16	0.11	0.21	0.16	0.12	0.12

Betweenness centrality

The measures presented so far encode the centrality of a node considering the connections with its neighbors. In some situations, the interactions between two non-adjacent nodes depend on the other vertices in the path between those two nodes [4]. Betweenness centrality aims to measure the influence of intermediate nodes considering the shortest paths between two ones in a graph. In this context, a node in a graph has a high centrality value, if it appears in many shortest paths connecting other nodes, as defined in Equation (S7):

$$c_i^B = \sum_{\substack{j=1 \\ j \neq i}}^{N} \sum_{\substack{k=1 \\ k \neq i,j}}^{N} \frac{n_{jk}(i)}{n_{jk}}, \quad i = 1, 2, \cdots, N. \tag{S7}$$

where N is the number of nodes, n_{jk} represents the number of the shortest paths from node j to node k and $n_{jk}(i)$ represents the number of the shortest paths from j to k that contain node i. For undirected graphs, the normalized Betweenness centrality is defined as:

$$nc_i^B = \frac{c_i^B}{(N-1)(N-2)/2} \tag{S8}$$

Tables S5 and S6 show the shortest paths and the numbers of n_{jk} and $n_{jk}(i)$, considering our example presented in Figure S1. Finally, Tables S7 and S8 show the absolute and normalized Betweenness centrality scores for different nodes.

Table S5: Shortest paths from node j to node k, i.e., n_{jk}.

n_{AB}	n_{AC}	n_{AD}	n_{AE}	n_{AF}	n_{AG}	n_{BC}	n_{BD}	n_{BE}	n_{BF}	n_{BG}	n_{CD}	n_{CE}	n_{CF}	n_{CG}	n_{DE}	n_{DF}	n_{DG}	n_{EF}	n_{EG}	n_{FG}
AB	ABC, ADC	AD	ADE	ADEF	ADEG	BC	BD	BDE	BDEF	BDEG	CD	CDE	CDEF	CDEG	DE	DEF	DEG	EF	EG	FG
1	2	1	1	1	1	1	1	1	1	1	1	1	1	1	1	1	1	1	1	1

Table S6: Shortest path from node j to node k that contains the node i (first row) and the division result between $n_{jk}(i)$ and n_{jk} (second row).

	A	B	C	D	E	F	G
Shortest paths	∅	ABC	∅	ADC;ADE;ADFE; ADEG;BDE;BDEF; BDEG;CDE;CDEF; CDEG	ADEF;ADFG;BDEF; BDEG;CDEF;CDEG; DEF; DEG	∅	∅
$\frac{n_{jk}(i)}{n_{jk}}$	0	1/2	0	1/2; 1; 1; 1; 1; 1; 1; 1; 1; 1	1; 1; 1; 1; 1; 1; 1; 1	0	0

Table S7: Betweenness centrality for the vertices in G.

c_A^B	c_B^B	c_C^B	c_D^B	c_E^B	c_F^B	c_G^B
0.0	0.5	0.0	9.5	8.0	0	0

Table S8: Normalized Betweenness centrality for the vertices in G.

c_A^B	c_B^B	c_C^B	c_D^B	c_E^B	c_F^B	c_G^B
0.0	0.033	0.0	0.633	0.533	0.0	0.0

References

[1] Mathieu Bastian, Sebastien Heymann, and Mathieu Jacomy. Gephi: An open source software for exploring and manipulating networks, 2009.

[2] M. E. J. Newman. *Networks: An Introduction.* Oxford University Press, 2010.

[3] Nicola Perra and Santo Fortunato. Spectral centrality measures in complex networks. *Phys. Rev. E*, 78:036107, Sep 2008.

[4] Gilbert Strang. *Introduction to Linear Algebra.* Wellesley-Cambridge Press, Wellesley, MA, fourth edition, 2009.

[5] Maarten van Steen. *Graph Theory and Complex Networks: An Introduction.* 2010.
